# Cardiovascular magnetic resonance evaluation of symptomatic severe aortic stenosis: association of circumferential myocardial strain and mortality

**DOI:** 10.1186/s12968-017-0329-7

**Published:** 2017-02-08

**Authors:** Tarique Al Musa, Akhlaque Uddin, Peter P. Swoboda, Timothy A. Fairbairn, Laura E. Dobson, Anvesha Singh, Pankaj Garg, Christopher D. Steadman, Bara Erhayiem, Ananth Kidambi, David P. Ripley, Adam K. McDiarmid, Philip Haaf, Daniel J. Blackman, Sven Plein, Gerald P. McCann, John P. Greenwood

**Affiliations:** 1 Multidisciplinary Cardiovascular Research Centre & The Division of Cardiovascular and Diabetes Research, Leeds Institute for Cardiovascular and Metabolic Medicine, University of Leeds, Leeds, LS2 9JT UK; 2 Department of Cardiovascular Sciences, and the National Institute of Health Research (NIHR), Cardiovascular Biomedical Research Unit, Glenfield General Hospital, University of Leicester, Leicester, UK; 3 Leeds Teaching Hospitals NHS Trust, Leeds General Infirmary, Leeds, UK

**Keywords:** Aortic stenosis, Transcatheter aortic valve implantation, Aortic valve replacement, Myocardial tissue tagging

## Abstract

**Background:**

It is unknown whether circumferential strain is associated with prognosis after treatment of aortic stenosis (AS). We aimed to characterise strain in severe AS, using myocardial tagging cardiovascular magnetic resonance (CMR), prior to and following Transcatheter Aortic Valve Implantation (TAVI) and Surgical Aortic Valve Replacement (SAVR), and determine whether abnormalities in strain were associated with outcome.

**Methods:**

CMR was performed pre- and 6 m post-intervention in 98 patients (52 TAVI, 46 SAVR; 77 ± 8 years) with severe AS. TAVI patients were older (80.9 ± 6.4 vs. 73.0 ± 7.0 years, p < 0.01) with a higher STS score (2.06 ± 0.6 vs. 6.03 ± 3.4, p < 0.001). Tagged cine images were acquired at the basal, mid and apical LV levels with a complementary spatial modulation of magnetization (CSPAMM) pulse sequence. Circumferential strain, strain rate and rotation were calculated using inTag© software.

**Results:**

No significant change in basal or mid LV circumferential strain, or of diastolic strain rate, was seen following either intervention. However, a significant and comparable decline in LV torsion and twist was observed (SAVR: torsion 14.08 ± 8.40 vs. 7.81 ± 4.51, p < 0.001, twist 16.17 ± 7.01 vs.12.45 ± 4.78, p < 0.01; TAVI: torsion 14.43 ± 4.66 vs. 11.20 ± 4.62, p < 0.001, twist 16.08 ± 5.36 vs. 12.36 ± 5.21, p < 0.001) which likely reflects an improvement towards normal physiology following relief of AS. Over a maximum 6.0y follow up, there were 23 (16%) deaths following valve intervention. On multivariable Cox analysis, baseline mid LV circumferential strain was significantly associated with all-cause mortality (hazard ratio, 1.03; 1.01–1.05; p = 0.009) independent of age, LV ejection fraction and STS mortality risk score. ROC analysis indicated a mid LV circumferential strain > −18.7% was associated with significantly reduced survival.

**Conclusion:**

TAVI and SAVR procedures are associated with comparable declines in rotational LV mechanics at 6 m, with largely unchanged strain and strain rates. Pre-operative peak mid LV circumferential strain is associated with post-operative mortality.

**Electronic supplementary material:**

The online version of this article (doi:10.1186/s12968-017-0329-7) contains supplementary material, which is available to authorized users.

## Background

Degenerative aortic valve stenosis (AS) is the commonest valvular disease in the western world currently affecting 3% of people over 75 years [1]. The left ventricle (LV) responds to pressure overload from aortic stenosis with hypertrophy to offset increased wall stress, in accordance with Laplace’s law [2]. This involves adverse remodelling of the extracellular matrix and altered protein composition which initially leads to a regional reduction in myocardial deformation, with global impairment in contraction occurring later [3].

Myocardial strain, strain rate and twist allow more sensitive characterisation of subtle myocardial performance [4] and can all be objectively quantified using myocardial tagging CMR with proven reproducibility [5, 6]. The myocardium deforms simultaneously in 3 directions and strain measurements can detect pathology prior to decline in conventional indices such as ejection fraction [7]. Patients with preserved cardiac output and severe AS reportedly exhibit compensatory high circumferential strain with increased apical rotation which are lost with decompensation of LV function [8].

Current guidelines recommend aortic valve replacement with the onset of symptoms or cardiac dysfunction (LVEF < 50%) [9]. However, impaired LVEF is a late change indicating significant myocardial damage and poorer outcomes, even after correction of AS [10]. It is notable LVEF is normal in most patients with severe AS, even when symptoms develop and that valve area and transvalvular gradients do not predict clinical outcomes following AVR [11]. The prognostic importance of circumferential myocardial strain in particular on outcome after treatment of aortic stenosis is unknown.

The aims of this study were to 1) characterise LV systolic and diastolic function as measured by CMR tagging in patients with severe symptomatic aortic stenosis prior to and following TAVI and SAVR, and 2) to assess whether CMR measures of strain could hold prognostic importance in those undergoing intervention.

## Methods

### Study population

146 patients were prospectively recruited with severe trileaflet degenerative AS who were referred for either TAVI (n = 91) or SAVR (n = 55) at the University Hospitals of Leeds and Leicester, UK, between July 2008 and December 2013. Severe AS was classified by transthoracic echocardiography (TTE) as an aortic valve area of ≤1.0 cm^2^ or peak velocity >4 m/s. The decision for TAVI or SAVR was taken by a multidisciplinary heart team in accordance with international guidance. Higher-risk (higher STS score) SAVR patients were recruited in preference to ensure baseline demographics were more comparable to the TAVI group. Exclusion criteria included any contraindication to CMR. Five age-comparable healthy controls were also scanned for comparison.

### Transcatheter aortic valve replacement

TAVI was performed under general anaesthesia. Either an 18 F CoreValve Revalving system (CVR, Medtronic, Minneapolis, Minnesota, USA) or an 18 or 20 F Lotus™ Aortic Valve system (Boston Scientific Corporation, Natick, MA, USA) were deployed as previously described [12, 13]. A transfemoral route was used preferentially when vascular access was suitable. In the presence of significant peripheral vascular disease, a subclavian artery approach was performed. The invasively measured LV end diastolic pressure was recorded from procedural details.

### Surgical aortic valve replacement

SAVR was performed by standard midline sternotomy with cardiopulmonary bypass and mild hypothermia. Biological or mechanical prostheses of varying sizes were used according to surgical preference; concomitant coronary artery bypass grafting was performed as indicated.

### CMR protocol

For each patient, identical preoperative and 6 m postoperative scans were performed on the same 1.5 T MRI vendor platform (Intera, Phillips Healthcare, Best, Netherlands or Avanto, Siemens Medical Systems, Erlangen, Germany). Both sites used the identical CMR protocol as previously described [14]; in brief this comprised standard steady-state free procession pulse sequences to image the entire left and right ventricle, through-plane velocity encoded phase contrast imaging to quantify aortic valve function and late gadolinium enhancement imaging 10 min after the administration of 0.2 mmol/kg of gadoteric acid to assess for fibrosis.

Complementary spatial modulation of magnetisation (CSPAMM) imaging was carried out during a single breath hold at end expiration in the short axis orientation, at the apex, mid-, and basal LV (multishot echo planar imaging, flip angle sweep applied to the radiofrequency excitation pulses of subsequent cardiac phases, two orthogonal line tags acquired per slice, field of view: 300 mm, matrix 128 × 128, slice thickness 10 mm, tag separation 8 mm, typically 18 phases, repetition time/echo time [TR/TE] 30 ms/6 ms, flip angle 25°). The “3 of 5 technique” was used to minimize variation in slice positioning between visits and has been demonstrated to be highly reproducible [5].

### Image analysis

All analysis was performed blinded off-line, using commercially available software (QMass 7.5 and QFlow 7.2, Medis Medical Imaging Systems, Leiden, The Netherlands). Standard ventricular and valvular assessment was performed as previously described [14]. Late gadolinium enhancement images were reviewed by two experienced observers for focal myocardial fibrosis and scarring (secondary to infarction) and reported qualitatively, as either present or absent, and then quantified using the full-width half-maximum technique.

CSPAMM analysis was performed for each myocardial slice using a dedicated tagging analysis package (inTag© software, Creatis, Lyon, Fr) and an example is depicted in an additional figure (see Additional file 1: Figure S1). Endocardial and epicardial contours were drawn for each slice, and a mid-myocardial contour was automatically calculated; contours were propagated through all cardiac phases. Strain is defined as an index that refers to the amount of myocardial deformation in one direction normalised to its initial dimension. Strain rate is the rate of deformation within in a time unit [2, 4]. Circumferential Lagrangian strain and strain rates (between epicardial and endocardial contours) and rotation were calculated for the three short axis slices. Left ventricular twist was determined as the difference between rotation at the apex and rotation at the base. Torsion was calculated as twist corrected for by length and radius of the LV cavity.

Intra-observer (12 data sets 6 months apart) and inter-observer (12 data sets) agreement was assessed using the intra-class correlation coefficient.

### Statistical analysis

Continuous variables are presented as mean ± SD. Normality was determined by the Shapiro–Wilk test. Frequencies are reported as number (%). The Student *t* test and Wilcoxon signed rank test were used to compare continuous variables as appropriate, and *χ*2 or Fisher’s exact test for categorical comparisons. Changes over time were assessed for differences between the treatment groups and clinical variables by two-way repeated measures analysis of variance (ANOVA). Cox proportional-hazards ratio regression analyses were performed to investigate univariate and multivariate correlates of all-cause mortality. Hazard ratios and 95% confidence intervals (CIs) were reported. Variables with univariate p < 0.05 were entered in the multivariate analysis in a stepwise forward approach. Receiver operating characteristic (ROC) curves were constructed to assess the sensitivity and specificity predictor variables. The cumulative event rates were calculated on the basis of the Kaplan-Meier method, and comparisons between groups were assessed by log-rank test. All statistical analyses were performed using the PASW software package (V.21.0 SPSS, IBM, Chicago, Illinois, USA) with the exception of ROC analysis that was performed with MedCalc version 9.3.1 (MedCalc Software, Mariakerke, Belgium). A two-sided significance level of p < 0.05 was considered statistically significant.

## Results

**Fig. 1:**
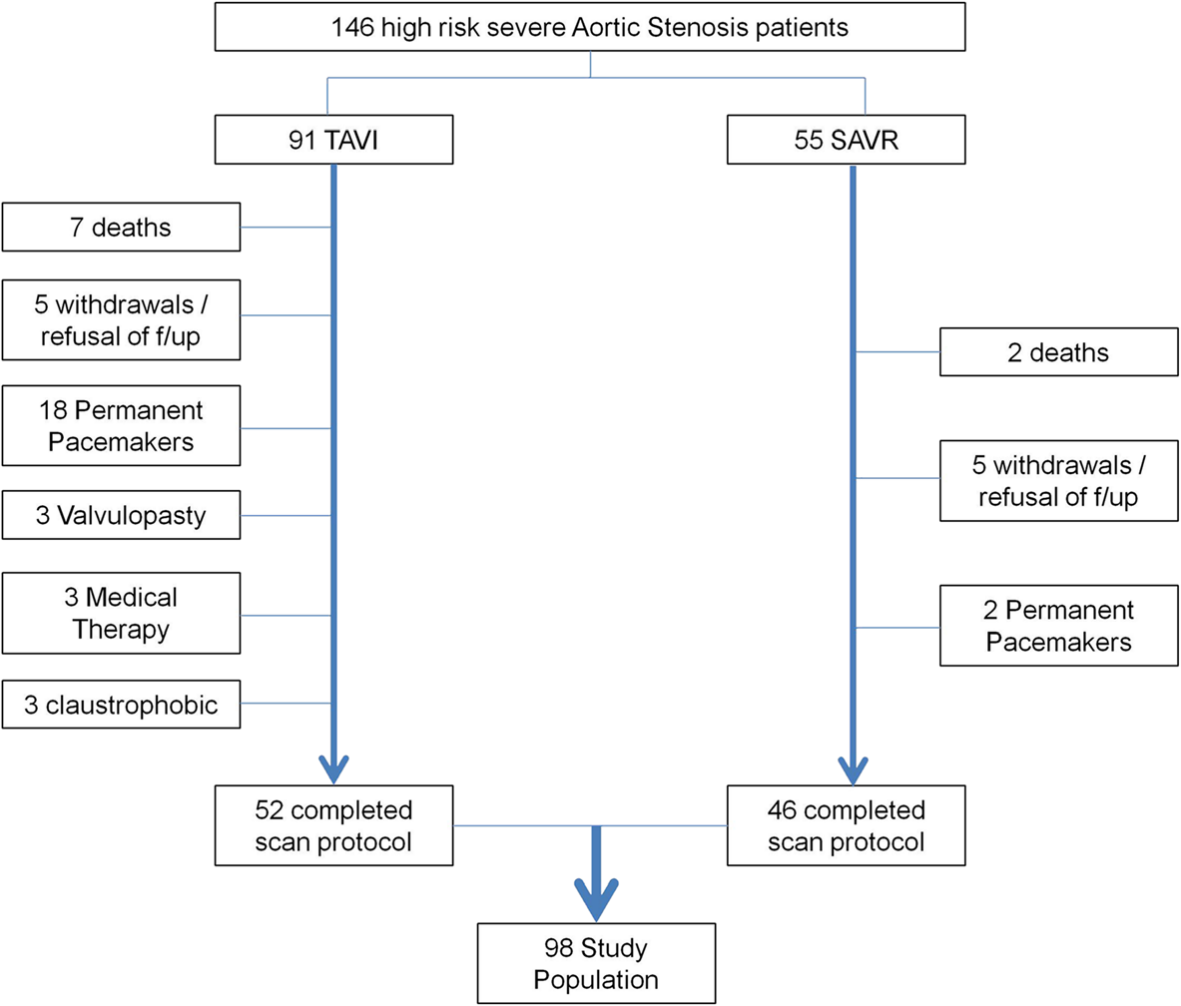
Study Profile

Ninety-eight patients (52 TAVI, 46 SAVR) with paired pre-operative and 6 m post-operative CMR scans were included for analysis. Reasons for non-completion of the CMR protocol were varied and are depicted in Fig. 1. At baseline, 15 patients did not undergo late gadolinium enhancement (LGE) imaging due to impaired renal function. Baseline characteristics of the study population are shown in Table 1 and grouped according to treatment received. As expected the TAVI group were older with a higher predicted 30 day mortality and greater frequency of prior coronary artery intervention.

**Table 1:** Patient characteristics and baseline echocardiographic data

Characteristics	SAVR (n = 46)	TAVI (n = 52)	p Value*
Age	73.0 ± 7.0	80.9 ± 6.4	<0.001
Male gender, n (%)	34 (74)	28 (54)	0.041
STS Mortality (%)	2.06 ± 0.6	6.03 ± 3.4	<0.001
BMI (kgm^−2^)	27.5 ± 4.4	27.0 ± 3.8	0.709
Previous MI, n (%)	6 (13)	9 (17)	0.560
Previous PCI, n (%)	1 (2)	14 (27)	0.001
Previous CABG, n (%)	1 (2)	17 (33)	<0.001
Stroke/TIA, n (%)	6 (13)	8 (15)	0.742
Peripheral vascular disease, n (%)	1 (2)	10 (19)	0.008
Hypertension	32 (70)	25 (48)	0.032
Diabetes Mellitus, n (%)	7 (15)	10 (19)	0.602
Hyperlipidaemia, n (%)	23 (50)	31 (60)	0.342
COPD, n (%)	4 (9)	12 (23)	0.056
Atrial Fibrillation, n (%)	3 (7)	15 (29)	0.005
eGFR (ml/min/1.73 m^2^)	73.3 ± 13.8	59.8 ± 18.9	<0.001
AVA (cm^2^)	0.83 ± 0..46	0.59 ± 0.17	<0.001
Mean aortic valve PG (mmHg)	46.9 ± 13.4	53.2 ± 19.2	0.102
Pulmonary Hypertension^a^, n (%)	7 (15)	15 (29)	0.110
ValvuloArterial Impedance (Z_va_)	3.73 ± 0.99	3.71 ± 1.09	0.734

Values are mean ± SD or n(%)

*AVA* aortic valve area, *CABG* coronary artery bypass grafting, *eGFR* estimated glomerular filtration rate, *COPD* chronic obstructive pulmonary disease, *MI* myocardial infarction, *MPG* mean pressure gradient, *NYHA* New York Health Association, *PCI* percutaneous coronary intervention, *Zva* valvuloarterial impedance (systolic arterial pressure + mean transvalvular gradient/stroke volume index)

*p Value for comparison between TAVI and SAVR groups

^a^Pulmonary hypertension defined as estimated pulmonary artery systolic pressure by transthoracic echocardiography to be >35 mmHg

### Measurement variability

Calculation of intra-class correlation coefficients indicated good intra- and inter-observer reproducibility of CMR measurements respectively: circumferential strain (0.98, 0.96) and LV twist (0.97, 0.95).

### Procedural data

Procedural data for TAVI and SAVR are summarised in an additional file (see Additional file 2: Table S1). For TAVI, 40(77%) patients received a Medtronic CoreValve and 12(23%) a Boston Scientific Lotus valve. A 29 mm valve was the most frequently used size (n = 26, 50%). In the surgical group, six patients received a mechanical prosthesis and the remaining 40(87%) a tissue bioprosthesis from various manufacturers. The modal valve size was 23 mm (n = 17, 37%). Twelve (26%) patients received concomitant coronary bypass grafting.

### Aortic Valve Haemodynamics and LV reverse remodelling

Results of the baseline and 6 m CMR scans are shown in Table 2. Significant reductions in peak aortic valve pressure gradient resulted in comparable reverse remodelling post-SAVR and TAVI; with reductions in both indexed LV EDV and mass.

**Table 2:** Preoperative baseline measurements and postoperative changes in the separate procedural groups

	SAVR		TAVI		p Value^†^
	Baseline	6 months	Baseline	6 months	
Haemodynamics					
Heart Rate (bpm)	63 ± 9	64 ± 12	67 ± 12	68 ± 15	0.952
Systolic BP (mmHg)	136 ± 21	133 ± 20	133 ± 25	137 ± 23	0.200
Diastolic BP (mmHg)	75 ± 10	72 ± 11	66 ± 10	65 ± 9	0.265
Valvular Function					
Aortic Peak PG (mmHg)^††^	52 ± 18	30 ± 13***	52 ± 16	22 ± 13***	0.072
Aortic Regurgitant Fraction (%)	19 ± 16	10 ± 11*	16 ± 11	9 ± 7**	0.936
MR fraction (%)	20 ± 18	8 ± 10**	26 ± 16	16 ± 16**	0.514
Left Ventricle					
EDVI (ml/m^2^)	94 ± 25	76 ± 13***	94 ± 21	88 ± 20**	0.023
LVEF (%)	58 ± 12	61 ± 11*	52 ± 11	54 ± 11	0.403
Mass Index (g/m^2^)	76 ± 22	61 ± 16***	79 ± 21	66 ± 18***	0.691
LVM/LVEDV (g/ml)	0.82 ± 0.2	0.81 ± 0.2	0.86 ± 0.2	0.77 ± 0.2***	0.041
Peak Circumferential strain					
Base (%)	−19.8 ± 5.2	−18.6 ± 4.9	−17.6 ± 5.8	−18.5 ± 5.8	0.03
Mid (%)	−21.1 ± 5.3	−20.0 ± 4.3	−19.3 ± 6.1	−19.8 ± 6.5	0.158
Apex (%)	−20.0 ± 6.4	−17.5 ± 6.8**	−18.8 ± 6.7	−18.8 ± 6.0	0.054
Peak mid-ventricular strain rate					
Systolic (S^−1^)	−0.032 ± 0.010	−0.034 ± 0.009	−0.029 ± 0.008	−0.032 ± 0.007**	0.188
Diastolic (S^−1^)	0.028 ± 0.016	0.028 ± 0.018	0.022 ± 0.015	0.023 ± 0.014	0.653
LV torsion	14.08 ± 8.40	7.81 ± 4.51***	14.43 ± 4.66	11.20 ± 4.62***	0.094
LV twist (°)	16.17 ± 7.01	12.45 ± 4.78**	16.08 ± 5.36	12.36 ± 5.21***	0.999

^†^Independent samples *t*-test to compare degree of change seen following SAVR with that seen following TAVI. Paired *t* test of baseline Vs 6 months: *p < 0.05, **p < 0.01, ***p < 0.001

### Measures of strain by CMR

At baseline, both groups undergoing SAVR and TAVI had comparable LV circumferential strain of the base (p = 0.081) mid (p = 0.128) and apex (0.318) with overall preserved LV ejection fraction. Similarly LV torsion (p = 0.845) and twist (p = 0.879) were comparable between groups. However, both systolic (p = 0.039) and diastolic (p = 0.037) strain rates were higher in the SAVR group.

At baseline for the TAVI group, there was moderate correlation between increasing LV end diastolic pressure (measured invasively during TAVI implantation) and both a deterioration in peak basal circumferential strain (r = 0.4, n = 33, p = 0.04, two-sided) and diastolic peak mid-ventricular strain rate (r = −0.5, n = 33, p = 0.003, two-sided).

No significant change in basal or mid-LV circumferential strain or of diastolic strain rate was seen following intervention, either post-SAVR or TAVI. A significant decline in peak apical circumferential strain following SAVR and an increase in circumferential systolic strain rate following TAVI were noted.

**Fig. 2:**
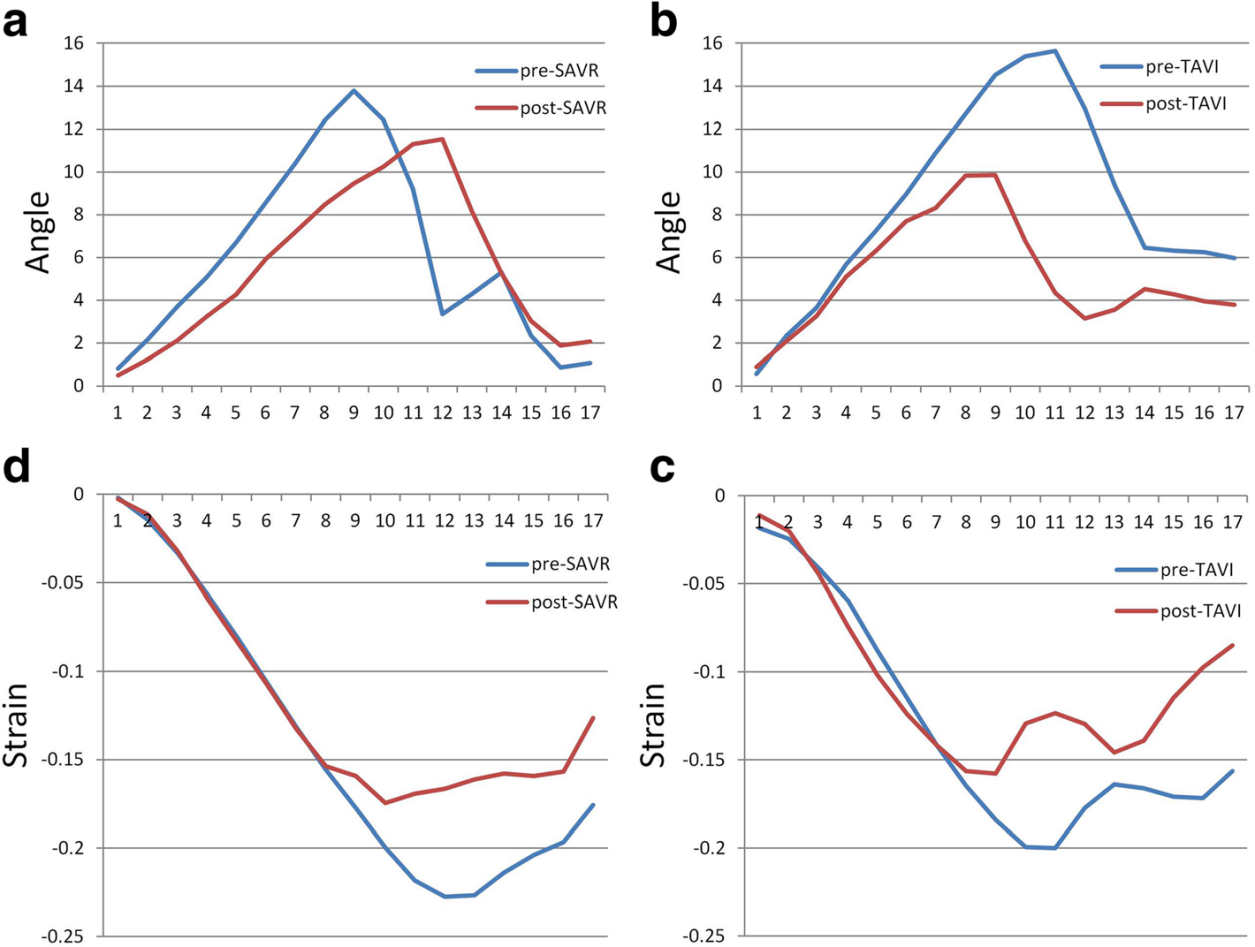
Change in twist and mid-LV circumferential strain. Twist pre and post-SAVR (**a**) and pre and post-TAVI (**b**). Circumferential strain pre and post-SAVR (**c**) and pre and post-TAVI (**d**)

Both SAVR and TAVI were associated with a significant and comparable decline in LV twist and torsion at 6 months following intervention (Fig. 2) consistent with normalisation towards physiological values (Table 3).

**Table 3:** Comparison of 6 month strain values following SAVR and TAVI with those from a healthy control group

	Control (n = 5)	SAVR 6 m (n = 46)	p Value	TAVI 6 m (n = 52)	p Value
Age (years)	72.6 ± 1.7	73.0 ± 7.0	0.769	80.9 ± 6.4	0.001
LVEF (%)	62.1 ± 6.0	61.1 ± 10.7	0.847	53.8 ± 10.5	0.092
LVMI (g/m^2^)	38.0 ± 7.1	60.8 ± 15.8	0.003	66.4 ± 17.9	0.001
LV torsion	12.58 ± 2.12	8.09 ± 4.85	0.047	11.20 ± 4.62	0.515
LV twist (°)	14.24 ± 2.58	12.68 ± 4.96	0.495	12.36 ± 5.21	0.432

Analysing the total severe AS patient population (n = 98), no change in LV strain at any level was seen following aortic valve intervention (Base: −0.186 ± 0.056 vs. −0.186 ± 0.054, p = 0.961; Mid: −0.201 ± 0.058 vs. −0.199 ± 0.006, p = 0.714; Apex: −0.194 ± 0.065 vs. −0.182 ± 0.064, p = 0.05).

### Predictors of mortality following intervention

**Fig. 3:**
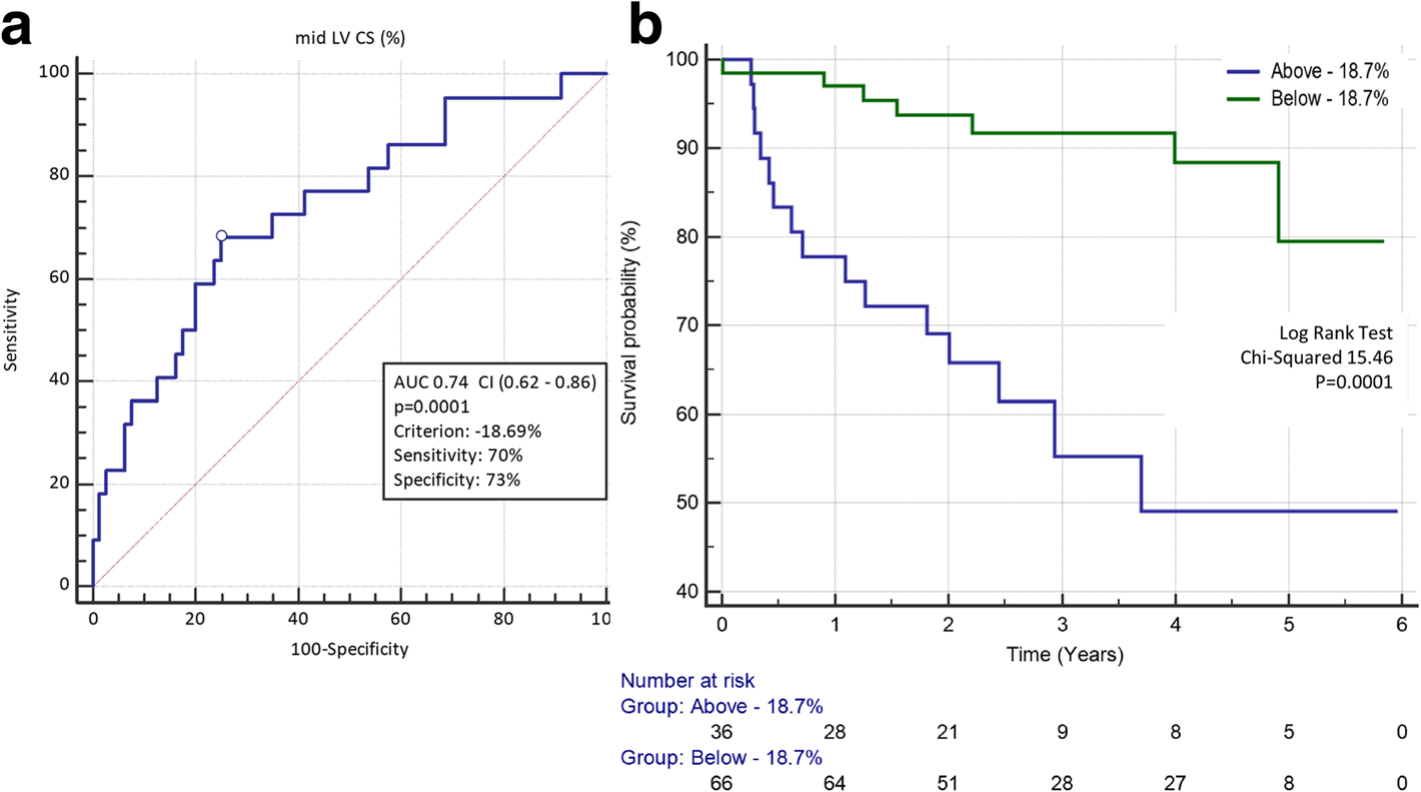
**a** ROC curve for baseline mid LV circumferential strain showing optimal discrimination value (−18.69%) and an AUC of 0.74. **b** Kaplan-Meier survival curves for AS patients undergoing valve intervention stratified by mid LV circumferential strain more positive than −18.7% (n = 67, *green*) or more negative than −18.7% (n = 40, *blue*). AUC: area under curve, CI: confidence interval

Over a maximum 6.0y follow up (median 2.5 years); there were 23 deaths (all-cause, of which 14 had completed follow-up imaging). Stepwise logistic regression identified a number of demographics and measures of cardiac function that were associated with mortality. Notably, the presence of baseline myocardial fibrosis (detected by LGE imaging), indexed LV mass, mean aortic pressure gradient and history of myocardial infarction were not significantly associated with prognosis. In multivariate analysis, baseline mid LV circumferential strain remained independently associated with all-cause mortality (Table 4). ROC analysis indicated the optimal threshold for pre-procedure mid LV circumferential strain to be −18.7%, from which a Kaplan-Meier graph was derived (Fig. 3).

**Table 4:** Cox proportional hazard analysis for prediction of all-cause death following valve intervention

Variable	Univariate analysis		Multivariate analyisis	
	Hazard ratio (95% CI)	p-Value	Hazard ratio (95% CI)	p-Value
Age (per year)	1.125 (1.043–1.215)	0.002	1.084 (0.935–1.256)	0.286
STS score (per %)	1.238 (1.037–1.477)	0.018	1.365 (0.943–1.976)	0.099
eGFR (per ml/min/1.73 m^2^)	0.963 (0.936–0.991)	0.010	0.988 (0.947–1.053)	0.953
Baseline Mid LV CS (per %)	1.016 (1.007–1.024)	0.001	1.029 (1.007–1.052)	0.009
Baseline LVEF (per %)	0.962 (0.926–0.999)	0.046	1.031 (0.949–1.119)	0.473
TAVI procedure	3.776 (1.283–11.109)	0.016	0.397 (0.043–3.646)	0.414
Myocardial Fibrosis (LGE + ve)	1.670 (0.615–4.541)	0.315	-	-
Baseline LVMI	1.014 (0.993–1.035)	0.189	-	-
History of MI	0.611 (0.148–2.516)	0.495	-	-
Mean Aortic PG	1.018 (0.91–1.046)	0.182	-	-
Indexed LA volume	1.012 (0.982–1.043)	0.428	-	-
LV mass:volume ratio	8.051 (0.332–195.380)	0.200	-	-
Zva	1.788 (0.847–3.778)	0.128	-	-
BMI	1.211 (0.990–1.482)	0.062	-	-
Diabetes Mellitus	1.119 (0.162–7.744)	0.909	-	-
Hyperlipidaemia	1.795 (0.396–8.138)	0.448	-	-
Hypertension	0.578 (0.112–2.994)	0.514	-	-
Previous MI	0.960 (0.136–6.778)	0.967	-	-
Previous PCI	0.733 (0.101–5.305)	0.758	-	-
Previous CABG	0.243 (0.043–1.385)	0.111	-	-
Atrial Fibrillation	0.980 (0.144–6.673)	0.984	-	-
Stroke/TIA	0.742 (0.069–7.960)	0.805	-	-
Peripheral Vascular Disease	1.589 (0.145–17.400)	0.705	-	-
COPD	0.907 (0.125–6.586)	0.924	-	-
Pulmonary Hypertension	0.692 (0.112–4.267)	0.692	-	-

## Discussion

This prospective multicentre study has demonstrated that in severe symptomatic AS patients with abnormal strain and torsion, a reduction in torsion but no recovery in circumferential strain is seen post-valve replacement (with either TAVI or SAVR). In addition, reduced baseline mid-LV circumferential strain was associated with a higher post-operative mortality, independent of age, STS predicted mortality and LVEF.

Previous studies in patients with symptomatic severe AS and preserved LV ejection fraction have reported uniformly reduced longitudinal strain [15–18]. Sustained severe AS culminates in hypertrophic LV remodelling and an elevation in LVEDP. This predisposes to subendocardial ischaemia and impairs longitudinal subendocardial fibre contractility [19] reducing global systolic function [20]. Our study is the first to define an inverse relationship between invasively measured LVEDP and CMR derived circumferential strain and diastolic strain rate in aortic stenosis.

Higher circumferential strain in patients with preserved LVEF, and increased apical rotation in patients with mild LV dysfunction are thought to indicate compensatory mechanics serving to maintain radial strain. These compensatory mechanisms are reduced as LV performance declines (8) and their loss appears to occur at the time of symptom onset [19] indicating their potential use for surveillance and timing of surgery [21].

At baseline, our severe AS population had abnormally low circumferential strain with normal twist values compared with echocardiography derived reference ranges [22]. This is indicative of failing dynamic LV compensation consistent with their symptomatic status, despite an overall preserved LVEF. Our findings are thus a novel contribution, distinct from prior published work [8, 23–25], in reporting the impact of treatment in a study population with more advanced LV dysfunction as a consequence of severe AS.

Our study population had particularly severe indices of AS severity and the observed reduced baseline strain may reflect diminished coronary flow reserve [23] and repetitive ischaemic myocardial injury [24]. Improved valvular function following intervention is thought to confer improved transmural myocardial perfusion and subsequent improved LV mechanics [26] but this was not seen in our study; neither following TAVI, SAVR, or as a treated AS population in its entirety. It is notable coronary heart disease is thought to blunt recovery of myocardial mechanics following SAVR [27]. In a sub analysis of our entire AS population excluding those with diseased epicardial vessels, we still failed to observe any improvement in circumferential strain at any LV level following treatment.

Conflicting changes in LV strain rates have been reported, with both improvements [17, 26, 28] and a decline (25) following SAVR; and either no change [24] or an improvement following TAVI [29]. Our study demonstrates neither SAVR nor TAVI was associated with improvement of peak circumferential strain at any level, or of diastolic strain rate. In a sub analysis of our entire AS population excluding those with late gadolinium enhancement, we still failed to observe any improvement in circumferential strain at any LV level following treatment. This lack of improvement indicates a degree of irreversible decompensation at baseline which may have potential future implications for surveillance of systolic function and timing of intervention in severe AS.

Our study provides unique insight into the assessment of LV rotational mechanics, which remains largely unaddressed by previous studies; both in the context of symptomatic AS and following aortic valve intervention. LV torsion and twist are integral components of ventricular contractility and diastolic filling [30]. Previous CMR studies have reported changes in rotation in the context of AS, but inferences were confounded by very small sample sizes (largest n = 13) [31, 32]. An increase in LV twist has been described in severe AS with preserved LVEF as compensation for impaired systolic longitudinal function [27]. The baseline LV twist in our patients was notably lower than those awaiting SAVR from published echo studies [33], again suggesting failure of compensation and a more advanced stage of disease [29].

Our study indicates that significant and comparable reductions in both torsion and twist, similar to that reported by others, occurs following SAVR [33] and TAVI [29]. Twist is an energy saving process reflecting the helical orientation of cardiac fibres which offsets afterload mismatch, generating high intra-cavity pressure with minimal fibre shortening [31]. In the context of AS and increased afterload, enhanced LV twist and torsion are observed to preserve adequate LV filling and untwisting [33–35]. Torsion is dependent on LV shape and falls with declining concentric hypertrophy, representing reduced leverage from epicardial fibres [36]. In our study, both TAVI and SAVR precipitated comparable reverse LV remodelling with significant reductions in both indexed LVEDV and LV mass. This is mechanistically consistent with our observed decline in twist and torsion which likely reflects an improvement in rotational mechanics towards more normal values.

Patients with severe aortic stenosis and reduced LV ejection fraction carry a high risk of mortality following both SAVR [37] and TAVI [38]. However, deteriorating ejection fraction is a late occurrence and significant interest remains in identifying advanced objective predictors of mortality when ejection fraction is above 50%. This study is, to our knowledge, the first to demonstrate circumferential strain derived by CMR is independently associated with all-cause postoperative mortality in symptomatic patients with severe AS and preserved LVEF undergoing intervention.

Previous studies have hypothesised the prognostic value of longitudinal strain in AS may reflect the impact of myocardial fibrosis and provide the link to poor outcome [39, 40]. This study has tested for an association between myocardial fibrosis and strain with respect to outcome, and demonstrates the prognostic importance of circumferential strain measurement is unrelated to late gadolinium enhancement. Lower circumferential strain in severe AS is independently associated with myocardial triglyceride accumulation [41]. It is possible this lipotoxicity, which is undetectable using conventional LGE imaging, is important to the link between strain and outcome. However, our findings are not fully explicable by myocardial steatosis which has been shown to regress following SAVR, albeit in younger patients than our study [41].

Based on our work, patients with severe AS, even in the context of preserved LVEF, are at high risk for mortality when baseline mid-LV circumferential strain is > −18.7%. It is noteworthy this association occurs despite the relief of index aortic stenosis with SAVR or TAVI, and thus these patients in particular may benefit from greater scrutiny in follow-up. Measurement of circumferential strain using CMR is a non-invasive and reproducible modality by which a single, breath-held acquisition can potentially provide prognostic information independent of age, LVEF and surgical risk score.

### Limitations

Our study population included patients with coronary artery disease and hypertension, rather than being restricted to pure aortic stenosis. This is however, generalisable to real world clinical practise and reduces the effect of selection bias. CMR assessment of cardiac rotational mechanics is sensitive to atrial fibrillation and regional wall motion abnormalities which can impair image quality. However, our quantification of strain using myocardial tagging CMR has demonstrated good reproducibility [5].

The control group comprised only 5 patients and ideally a larger sample would have facilitated a more robust comparison. There is paucity in the literature in regards to normal CMR strain ranges particularly in patients aged 80 years and above as it is challenging to recruit volunteers with structurally normal hearts from this age group.

Our study population size is small with relatively few events carrying a risk of potential statistical over fitting. Also, we have reported all-cause mortality rather than cardiac mortality. Thus our findings need to be viewed with caution and validated in larger outcome studies. The regression analysis was aimed at identifying factors associated with persistently poor outcomes *after* aortic stenosis has already been relieved; thus our results relate to a specific patient population and should not be over-generalised. Finally, we only enrolled patients with symptomatic aortic stenosis and further work is required to determine whether the prognostic importance of strain assessment can be extended to those who are asymptomatic; and thus potentially influence surgical timing.

## Conclusions

Patients with symptomatic severe AS and preserved LVEF undergoing aortic valve intervention have reduced peak circumferential strain and systolic strain rates. At 6 m, TAVI and SAVR procedures were associated with comparable declines in rotational LV mechanics, with largely unchanged strain and strain rates. Pre-operative peak mid LV circumferential strain was associated with post-operative total mortality and requires further investigation as to its use as a risk stratification tool.

## Additional files

Additional file 1:
**Figure S1.** Example of inTag© analysis using CSPAMM. (DOCX 81 kb)
Additional file 2:
**Table S1.** Procedural and operative data for TAVI and SAVR procedures. (DOCX 30 kb)

## Abbreviations

AR: Aortic regurgitation
AS: Aortic stenosis
AVA: Aortic valve area
BMI: Body mass index
CABG: Coronary artery bypass grafting
CMR: Cardiovascular magnetic resonance
COPD: Chronic obstructive pulmonary disease
CSPAMM: Complementary spatial modulation of magnetisation
eGFR: estimated glomerular filtration rate
EuroSCORE: European System for Cardiac Operative Risk Evaluation
LGE: Late gadolinium enhancement
LVEDP: Left ventricular end diastolic pressure
LVEF: Left ventricular ejection fraction
LVM: Left ventricular mass
MI: Myocardial infarction
MPG: Mean pressure gradient
MR: Mitral regurgitation
NYHA: New York Heart Association
PCI: Percutaneous coronary intervention
ROC: Receiver operating characteristic
SAVR: Surgical aortic valve replacement
STS: Society of Thoracic Surgeons’ risk models
TAVI: Transcatheter aortic valve implantation
Zva: Valvuloarterial impedance
