# Supplementary material for: Cardiovascular magnetic resonance evaluation of symptomatic severe aortic stenosis: association of circumferential myocardial strain and mortality
Source: J Cardiovasc Magn Reson. 2017 Feb 8;19:13. doi: 10.1186/s12968-017-0329-7 (PMC5297161; doi:10.1186/s12968-017-0329-7)

**Additional file 1: Figure S1:**  Example of inTag© analysis using complementary spatial modulation of magnetization (CSPAMM). Diastole (A, C) and systole (B, D).


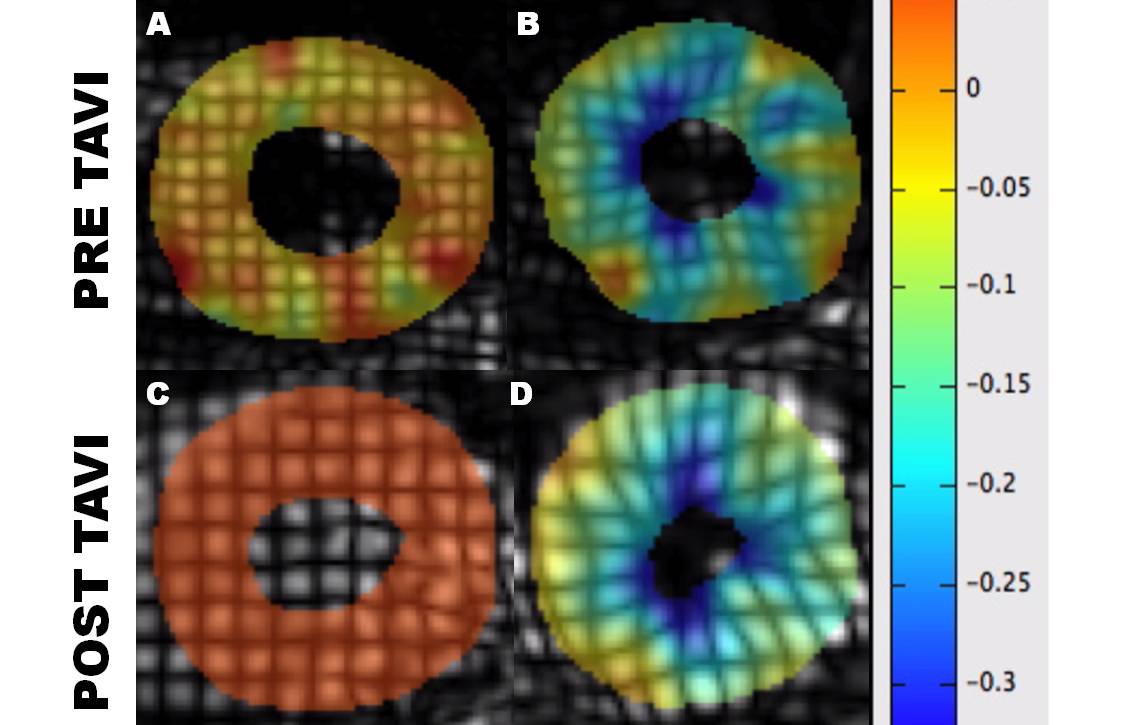

Supplement: Additional file 1: — Figure S1. Example of inTag© analysis using CSPAMM. (DOCX 81 kb) [file 12968_2017_329_MOESM1_ESM.docx]
