# Supplementary material for: Cardiovascular magnetic resonance evaluation of symptomatic severe aortic stenosis: association of circumferential myocardial strain and mortality
Source: J Cardiovasc Magn Reson. 2017 Feb 8;19:13. doi: 10.1186/s12968-017-0329-7 (PMC5297161; doi:10.1186/s12968-017-0329-7)
Supplement: Additional file 2: — Table S1. Procedural and operative data for TAVI and SAVR procedures. (DOCX 30 kb) [file 12968_2017_329_MOESM2_ESM.docx]

**Additional file 2: Table S1:** Procedural and operative dataTAVI: transcatheter aortic valve implantation, SAVR: surgical aortic valve replacement, CABG: coronary artery bypass grafting, LIMA: left anterior descending artery.
